# Vanadium Dioxide Nanocoating Induces Tumor Cell Death through Mitochondrial Electron Transport Chain Interruption

**DOI:** 10.1002/gch2.201800058

**Published:** 2018-12-03

**Authors:** Jinhua Li, Meng Jiang, Huaijuan Zhou, Ping Jin, Kenneth M. C. Cheung, Paul K. Chu, Kelvin W. K. Yeung

**Affiliations:** ^1^ Department of Orthopaedics and Traumatology Li Ka Shing Faculty of Medicine The University of Hong Kong Pokfulam Hong Kong 999077 China; ^2^ Department of Physics and Department of Materials Science and Engineering City University of Hong Kong Tat Chee Avenue Kowloon Hong Kong 999077 China; ^3^ Centre for Translational Bone Joint and Soft Tissue Research University Hospital Carl Gustav Carus and Faculty of Medicine Technische Universität Dresden Dresden 01307 Germany; ^4^ Shenzhen Key Laboratory for Innovative Technology in Orthopaedic Trauma Department of Orthopaedics and Traumatology The University of Hong Kong‐Shenzhen Hospital Shenzhen 518053 China; ^5^ College of Medical Imaging Shanghai University of Medicine and Health Sciences Shanghai 201318 China; ^6^ State Key Laboratory of High Performance Ceramics and Superfine Microstructure Shanghai Institute of Ceramics Chinese Academy of Sciences Shanghai 200050 China

**Keywords:** anticancer, charge transfer, functional coatings and films, mitochondria, vanadium dioxide

## Abstract

A biomaterials surface enabling the induction of tumor cell death is particularly desirable for implantable biomedical devices that directly contact tumor tissues. However, this specific antitumor feature is rarely found. Consequently, an antitumor‐cell nanocoating comprised of vanadium dioxide (VO_2_) prepared by customized reactive magnetron sputtering has been proposed, and its antitumor‐growth capability has been demonstrated using human cholangiocarcinoma cells. The results reveal that the VO_2_ nanocoating is able to interrupt the mitochondrial electron transport chain and then elevate the intracellular reactive oxygen species levels, leading to the collapse of the mitochondrial membrane potential and the destruction of cell redox homeostasis. Indeed, this chain reaction can effectively trigger oxidative damage in the cholangiocarcinoma cells. Additionally, this study has provided new insights into designing a tumor‐cell‐inhibited biomaterial surface, which is modulated by the mechanism of mitochondria‐targeting tumor cell death.

## 1 Introduction

Implantable biomedical devices, such as orthopedic implants, catheters, and stents, have been widely adopted to treat many terminal diseases to reduce patients' pain and improve their quality of life.^1^ For instance, the current treatment protocol for patients suffering with osteosarcoma consists of surgical removal of bone tumors and reconstruction of bone defects through orthopedic implantation.^2^ However, surgical intervention is not able to entirely remove osteosarcoma cells, and therefore postoperation chemotherapy or radiotherapy is usually necessary to eliminate the residual tumor cells.^3^ To decrease the viability of tumor cells on the implant surface, researchers have proposed modifying the biomaterial surface with antitumor coatings.^4^ Cholangiocarcinoma is usually fatal and notoriously hard to diagnose because its clinical presentation occurs after it is already too late to successfully treat.^5^ Malignant biliary obstruction is the primary cause of the high mortality rate of cholangiocarcinoma.^6^ The surgical insertion of biliary stents to assist in biliary drainage can effectively reduce mortality and morbidity.^7^ However, the primary clinical deficiency of this long‐term stenting is frequent clogging due to tumor cell growth,^8^ which suggests that elimination of the tumor growth could effectively restore stent potency by allowing better biliary drainage.

The strategy most often adopted to inhibit tumor cell growth on implants is to modify the biomaterials surface using anticancer coatings that release drugs or ions locally.^9^ In recent years, nanoparticle‐based anticancer agents have been widely explored^10^ and represent a paradigm shift in terms of inhibiting tumor cell growth on biomaterial implants. Fe_3_O_4_,^11^ Mn_3_O_4_,^12^ ZnO,^13^ CeO_2_,^14^ and CuO^15^ are the most commonly used anticancer nanoparticles. Vanadate‐based upconversion nanoparticles have attracted considerable attention in biomedical imaging and theranostic applications.^16^ Many researchers have realized the potential of vanadium to reduce or even prevent tumor cell growth. Studies have mainly focused on organic vanadium coordination compounds (vanadocene acetylacetonate, vanadocene dichloride, etc.) and inorganic vanadium salts (Na_3_VO_3_, VOSO_4_, etc.),^17^ and the active center of these compounds is the released pentavalent (+5) vanadate or tetravalent (+4) vanadyl ions. However, there is a risk of lowering blood glucose levels with direct oral administration or injection of these soluble vanadium compounds when chemotherapy is applied.^18^ Therefore, the slow release of vanadium ions directly from the biomaterial surface has been considered for clinical applications.

In order to resist the growth of tumor cells on the implant surface, we propose the construction of a vanadium‐containing nanocoating on the surface that can inhibit or kill tumor cells through reactive oxygen species (ROS) overproduction induced by the controlled release of vanadium ions. Trivalent (+3), tetravalent (+4), and pentavalent (+5) vanadium are the most common forms, and their respective oxides are V_2_O_3_, VO_2_, and V_2_O_5_.^19^ Recently, the intermediate oxide VO_2_ has demonstrated antibacterial and osteogenic potential,^20^ and the biofunction of vanadium is dose dependent.[[qv: 18b]] We hypothesize that this functional surface with a tailor‐made VO_2_ nanocoating can effectively direct tumor cell fate, thereby inducing tumor cell death.

To realize the proposed concept, a customized magnetron sputtering technique has been utilized to fabricate the VO_2_ nanocoating, and the film thickness has been optimized by tuning the deposition time. In brief, we fabricated a VO_2_ nanocoating on quartz glass through the reactive sputtering of vanadium metal with O_2_ gas. Following the characterization of the physical and chemical properties of the VO_2_‐modified quartz surface, the inhibition effect of this surface was tested by culturing human cholangiocarcinoma cells. Meanwhile, the cytocompatibility of this surface was also tested by culturing rat bone marrow mesenchymal stem cells. Furthermore, the detailed biological responses and underlying mechanism of tumor cell inhibition were investigated by the coincubation of glutathione antioxidant on the VO_2_‐modified surface.

## 2 Results

### 2.1 Sample Characterization and Analysis

**Figure 1:**
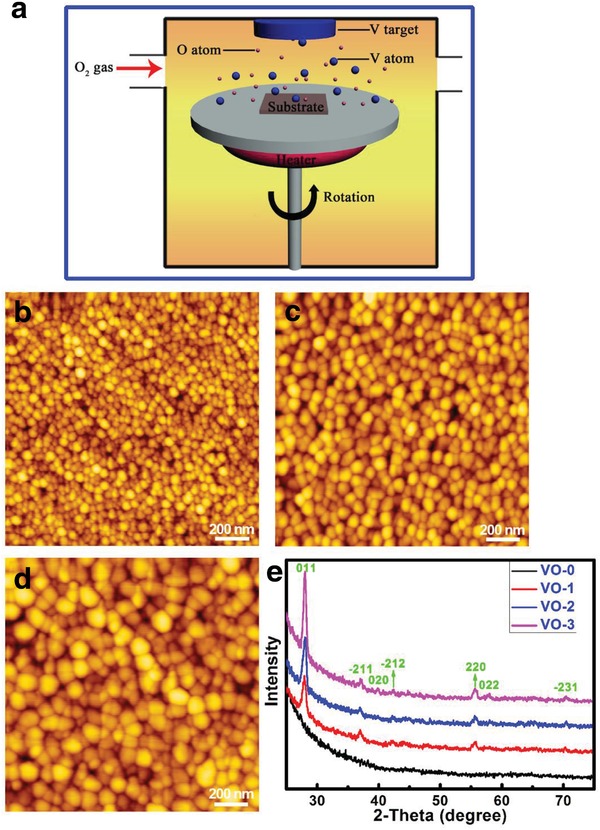
a) Schematic illustration for the deposition of VO_2_ nanofilm on substrate synthesized by reactive sputtering of metallic V target with O_2_ gas introduction under a heating condition. AFM images of the surfaces of b) VO‐1, c) VO‐2, and d) VO‐3 samples. e) XRD patterns of the samples VO‐0, VO‐1, VO‐2, and VO‐3.

**Figure** **1**a illustrates the fabrication scheme for the VO_2_ nanocoating on the substrate through reactive magnetron sputtering of the V metal target and O_2_ gas. Figure [qv: 1]b–d shows the surface atomic force microscopy (AFM) images of the VO_2_ samples on quartz glass. With the help of the AFM tapping mode, the VO‐1 sample (30 nm thickness) was seen to have a homogeneous compact film morphology consisting of tiny nanoparticles (Figure 1b). As for the VO‐2 sample (80 nm thickness), the size of the nanoparticles was larger, as seen in Figure 1c, and this was also true for the nanoparticles on the VO‐3 sample (120 nm thickness, Figure 1d). In consequence, prolonging the deposition time increased the nanoparticle size, which was ascribed to the Volmer–Weber growth mode of VO_2_ thin films. Combined with the X‐ray diffractometer (XRD) 2θ scan profiles in Figure 1e, only the characteristic diffraction peaks of the monoclinic VO_2_ phase were detected in the XRD pattern. All of the positions of the diffraction peaks were accordant with the standard reference data (JCPDS No. 72–0514), demonstrating the exclusive generation of single‐phase VO_2_ polycrystalline films. Meanwhile, the increase in thickness resulted in better crystallinity for the VO_2_ films, as evidenced by the rising intensity of the diffraction peaks. Additionally, the full width at half maxima (FWHM) values of the dominant orientation (011) were reduced as the VO_2_ film thickness increased. Based on the Scherrer formula, the average grain size of the VO_2_ films had a positive correlation with the reciprocal of the FWHM, which thus provided more support to explain the growing nanoparticle size.^21^

**Figure 2:**
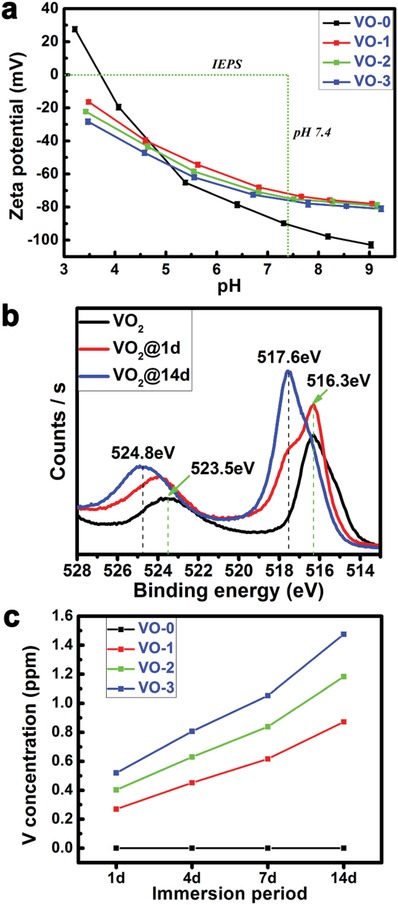
a) Measurement results of surface zeta potentials of samples VO‐0, VO‐1, VO‐2, and VO‐3. b) Chemical valence states of V element before and after soaking in PBS for 1 and 14 days. c) Release profiles of vanadium ions from samples VO‐0, VO‐1, VO‐2, and VO‐3 within 14 days.

The surface zeta potentials of the samples are given in **Figure** **2**a. The isoelectric point of a surface (IEPS) is the pH of an aqueous solution when the total surface charge density is zero, and a substrate surface is negatively (positively) charged, since pH is higher (lower) than IEPS.^22^ For VO‐0, at the IEPS (3.7), the total charge density of the hydrated surface on quartz was zero (SiO_2_ + H_2_O = H_2_SiO_3_). For VO_2_ samples, intermediate state VO_2_ tends to oxidize to V_2_O_5_ (4VO_2_ + O_2_ → 2V_2_O_5_), and the total charge density of the hydrated surface is zero at IEPS (V_2_O_5_ + 3H_2_O = 2H_3_VO_4_). Here, a negatively charged surface was generated on VO_2_ thin films in a wide pH range because of the p*K*_a_ values for vanadate (H_3_VO_4_ + H_2_O ↔ H_2_VO_4_^−^ + H_3_O^+^, p*K*_a_ = 3.5; H_2_VO_4_^−^ + H_2_O ↔ HVO_4_^2−^ + H_3_O^+^, p*K*_a_ = 7.8; HVO_4_^2−^ + OH^−^ ↔ VO_4_^3−^ + H_2_O, p*K*_a_ = 12.5).^23^ In the present study, the VO‐3 samples were immersed in phosphate buffered saline (PBS) for 1 and 14 days, respectively. Then, X‐ray photoelectron spectroscopy (XPS) was used to determine the chemical valence states of the vanadium element. As seen in Figure 2b, the XPS doublet peaks at 516.3 and 523.5 eV belonged to the V2p_3/2_ and V2p_1/2_ in VO_2_, respectively.^24^ After one day of immersion in PBS, the surface partially oxidized, with an obvious positive shift in the binding energy. After soaking in PBS for 14 days, the doublet peaks of V2p_3/2_ (517.6 eV) and V2p_1/2_ (524.8 eV) in V_2_O_5_ were the major peaks detected on the surface.^25^ Therefore, the zeta potential and XPS analysis results were consistent. Meanwhile, the release profiles of the vanadium ions from the VO_2_ nanocoating in RPMI 1640 medium are shown in Figure 2c. In the figure, it can be seen that vanadium ions were released from the VO_2_ nanocoating in a time‐dependent and thickness‐dependent manner, showing the intended difference among groups.

### 2.2 Tumor Cell Growth and Viability

**Figure 3:**
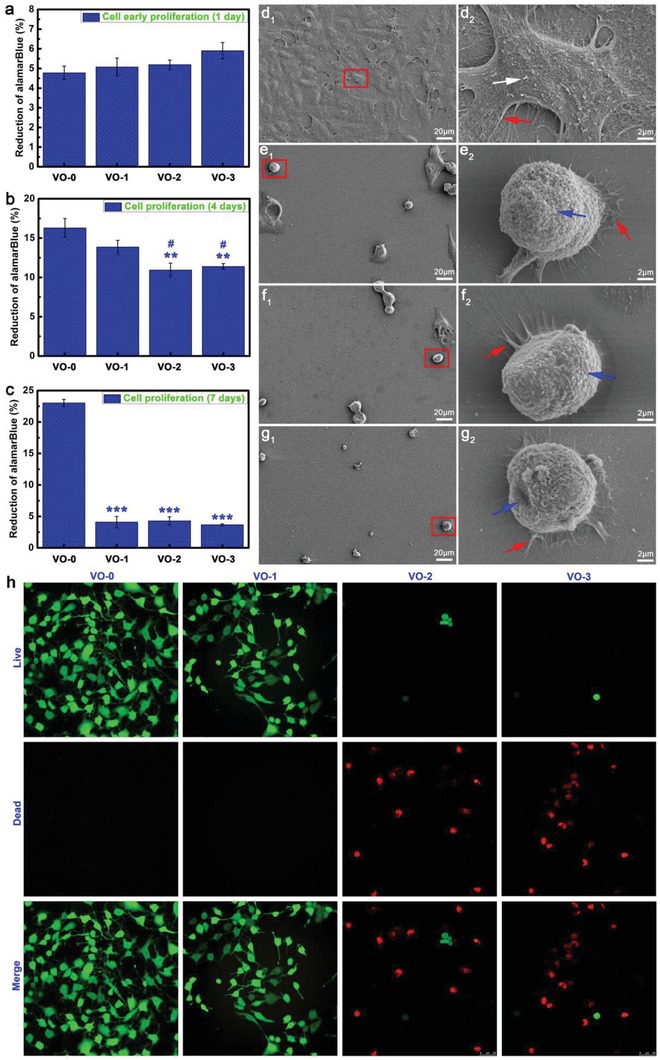
Tumor cell viability results after a) 1 day, b) 4 days, and c) 7 days of culture on the samples VO‐0, VO‐1, VO‐2, and VO‐3 (Note: **P < 0.01, ***P < 0.001 versus VO‐0; ^#^ *P* < 0.05 versus VO‐1). Tumor cell morphology and cell–material interactions after 4 days of culture on the samples d_1_,d_2_) VO‐0, e_1_,e_2_) VO‐1, f_1_,f_2_) VO‐2, and g_1_,g_2_) VO‐3 (Note: white arrow, indicating the exuberant extracellular matrix mineralization; red arrow, indicating the abundant lamellipodia and filopodia extensions; blue arrow, indicating the deteriorating cell membrane). h) Live/dead fluorescence staining results of tumor cells after 4 days of culture on the samples VO‐0, VO‐1, VO‐2, and VO‐3.

The early cell proliferation at day one corresponds to the initial cell adhesion that occurs within 24 h.^26^ As shown in **Figure** **3**a, at day one of early culture, the tumor cells can adhere to the VO_2_ nanocoating with an alamarBlue reduction rate around 5%, showing no significant difference with the VO‐0 quartz control sample. However, after four days of culturing (Figure 3b), the tumor cell proliferation appeared to be inhibited on the VO_2_ nanocoating due to vanadium exposure, and the reduction rates of alamarBlue were in the following order: VO‐0 (16.3 ± 1.2%) > VO‐1 (13.9 ± 0.8%) > VO‐2 (10.9 ± 0.9%) ≈ VO‐3 (11.4 ± 0.4%). At day seven of culture (Figure 3c), the tumor cells proliferated well on the VO‐0 surface, and the reduction rate of alamarBlue was higher than 22.5%, while the reduction rates for the VO_2_ nanocoating samples were lower than 5%. This could indicate that the tumor cells were suffering apoptosis on all of the VO_2_‐nanocoated samples. The observed tumor cell inhibition and toxicity exhibited a dose‐dependent and time‐dependent effect. According to the results, the presence of a VO_2_ nanocoating on quartz glass could cause severe cytotoxicity to tumor cells.

Scanning electron microscopy (SEM) was performed to analyze the tumor cell morphology development and cell–material interactions after four days of culturing on the surfaces of VO_2_‐nanocoated and quartz substrates. As seen in Figure 3d1, the surface of the VO‐0 quartz glass was thoroughly covered by the tumor cells, and the cells appeared to connect well with each other, indicating their healthy state. On the contrary, the number of tumor cells was significantly reduced on the VO_2_ nanocoating, and most of them maintained a spherical or spindle morphology (Figure 3e1,f_1_,g_1_). Consequently, the cell morphology observations were very accordant with the cell proliferation trend. At high magnification, the tumor cells spread well on the VO‐0 quartz glass, showing a multipolar spindle morphology (Figure 3d2). Focusing on the outer cell membrane, visible particulate matter was prevalent (white arrow), indicating the exuberant extracellular matrix mineralization. Furthermore, abundant lamellipodia and filopodia extensions could be seen as well (red arrow), indicating the pervasive intercellular communications and cell–material interactions. However, the spherical cell morphology along with a deteriorating cell membrane (blue arrows in Figure 3e2,f_2_,g_2_) were predominant in the tumor cells cultured on the VO_2_ nanocoating, indicating the poor cellular viability and apoptosis of the tumor cells. Interestingly, the lamellipodia and filopodia, which are indicated by the red arrows in Figure 3e2,f_2_,g_2_ showed an initial cell adhesion, spreading, and cell–material interactions, which was in agreement with the analysis of Figure 3a–c. Meanwhile, visualization of the tumor cell viability in Figure 3h shows the live/dead fluorescence staining results, which reveal that the number of live cells (stained in green fluorescence) reduced sharply on the VO_2_ nanocoating at day four of culture. Moreover, increasing number of dead cells (stained in red fluorescence) were observed, further demonstrating that exposure to the VO_2_ nanocoating caused a severe decrease in the viability of tumor cells, especially in the VO‐2 and VO‐3 samples.

### 2.3 Intracellular Reactive Oxygen Species Production

**Figure 4:**
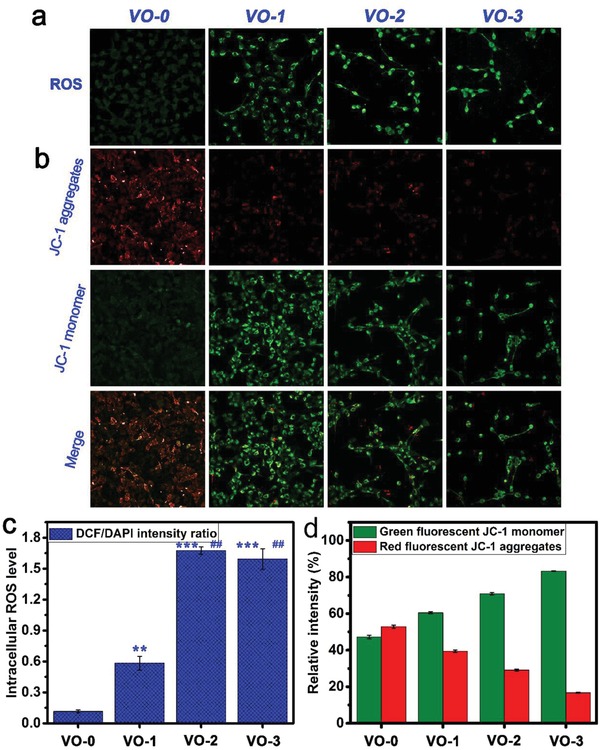
Fluorescence staining images of a) intracellular ROS for tumor cells and b) mitochondria membrane potentials after 4 days of culture on the samples VO‐0, VO‐1, VO‐2, and VO‐3, accompanied by the corresponding quantitative results of c) ROS levels and d) membrane potentials. Note: **P < 0.01, ***P < 0.001 versus VO‐0; ^##^ *P* < 0.01 versus VO‐1.

The intracellular ROS levels were investigated qualitatively and quantitatively. First, ROS fluorescence staining was performed using 2′,7′‐dichlorodihydrofluorescein diacetate (DCFH‐DA) to visualize the intracellular ROS level. As shown in **Figure** **4**a, the intensities of the 2′,7′‐dichlorofluorescein (DCF) green fluorescence, indicating intracellular ROS levels for tumor cells cultured on the VO_2_ nanocoating, were significantly stronger than those on VO‐0, and the levels correlated with the film thickness in a dependent manner, indicating that the intracellular ROS level was elevated in thicker films. Figure 4c shows the corresponding intracellular ROS level expressed by the ratios of DCF and 4′,6‐diamidino‐2‐phenylindole (DAPI) fluorescence intensities. Quantitatively, exposure to the VO_2_ nanocoating could obviously elevate the intracellular ROS levels of tumor cells, and the order of DCF/DAPI intensity ratios was as follows: VO‐3 (1.6 ± 0.1) ≈ VO‐2 (1.7 ± 0.03) > VO‐1 (0.6 ± 0.1) > VO‐0 (0.1 ± 0.01). Consequently, the ROS level obviously exceeds the threshold that cells can tolerate to maintain redox homeostasis, causing oxidative damage.

### 2.4 Mitochondrial Membrane Potentials

**Figure 5:**
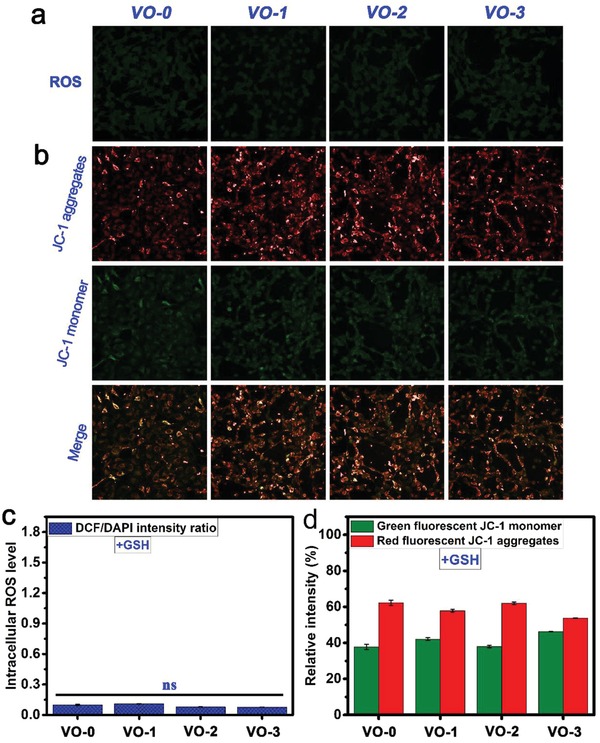
Fluorescence staining images of a) intracellular ROS for tumor cells and b) mitochondria membrane potentials after 4 days of culture on the samples VO‐0, VO‐1, VO‐2, and VO‐3 with adding GSH, accompanied by the corresponding quantitative results of c) ROS levels and d) membrane potentials. Note: ns, not significant.

**Figure 6:**
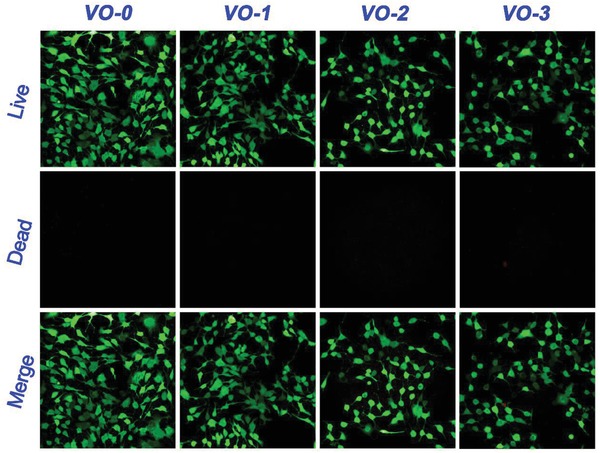
Live/dead fluorescence staining results of tumor cells after 4 days of culture on the samples VO‐0, VO‐1, VO‐2, and VO‐3 with adding GSH.

The mitochondrial membrane potentials (ΔΨ_m_) of the tumor cells cultured on various samples were detected using 5,5′,6,6′‐tetrachloro‐1,1′,3,3′‐tetraethyl benzimidazolyl carbocyanine iodide (JC‐1) dye, both qualitatively and quantitatively. A high ΔΨ_m_ facilitates the generation of JC‐1 aggregates that fluoresce red in the mitochondrial matrix, while a low ΔΨ_m_ promotes the presence of JC‐1 monomers, which fluoresce green, in cytoplasm. Figure 4b shows the representative images of the JC‐1 assay on tumor cells after VO_2_‐nanocoating exposure. For unexposed tumor cells on VO‐0, well‐polarized mitochondria were stained in bright red and light green fluorescence. By contrast, the VO_2_‐nanocoating exposure caused significant changes in the tumor cell staining: the red fluorescence gradually decreased while the green fluorescence obviously increased. These changes in JC‐1 fluorescence following VO_2_‐nanocoating exposure indicated a reduction in the JC‐1 aggregates that accumulated in well‐polarized mitochondria and the diffusion of JC‐1 monomers into the cytoplasm from depolarized mitochondria, further demonstrating the collapse of the mitochondrial membrane potentials and the increase in the mitochondrial membrane permeability. Quantitatively, as seen in Figure 4d, the relative fluorescence intensities of JC‐1 aggregates (red) and JC‐1 monomers (green) showed no obvious differences for tumor cells cultured on VO‐0. However, after VO_2_‐nanocoating exposure, the gap between the red fluorescence decrease and green fluorescence increase gradually became larger in a film thickness‐dependent manner. The relative intensities (%) of red fluorescence were in the following order: VO‐0 (52.8 ± 0.9%) > VO‐1 (39.5 ± 0.6%) > VO‐2 (29.1 ± 0.6%) > VO‐3 (16.7 ± 0.2%). Accordingly, the order of the relative intensities (%) of green fluorescence was as follows: VO‐0 (47.2 ± 0.9%) < VO‐1 (60.5 ± 0.6%) < VO‐2 (70.9 ± 0.6%) < VO‐3 (83.3 ± 0.2%). This indicated the status of the ΔΨ_m_ collapse and depolarization in the mitochondria of tumor cells upon VO_2_‐nanocoating exposure, which was consistent with the qualitative observations. The collapse of the ΔΨ_m_ is an indicator of mitochondria depolarization, impairment, and dysfunction and is an early mark of cell apoptosis.^27^ **Figure** **5** shows the qualitative and quantitative results of intracellular ROS levels and mitochondrial membrane potentials for the tumor cells cultured on various samples with coincubation of glutathione antioxidant. As seen in Figure 5a, the DCF green fluorescence intensities showed no significant differences among groups, which was further demonstrated by the quantitative results in Figure 5c, showing DCF/DAPI intensity ratios lower than 0.15. This indicated that no significant differences existed in the intracellular ROS levels for the cultured tumor cells on various samples with glutathione coincubation. The results were similar for the mitochondrial membrane potentials. As shown in Figure 5b, with glutathione coincubation, the well‐polarized mitochondria of tumor cells cultured on both VO‐0 and VO_2_ nanocoating samples were stained in bright red and light green fluorescence. The relative intensities (%) of red fluorescence and green fluorescence were around 60% and 40%, respectively, showing no significant differences among groups (Figure 5d). Therefore, the coincubation of glutathione antioxidant can effectively alleviate intracellular ROS production, mitochondrial membrane potential collapse, and depolarization of tumor cells upon exposure to the VO_2_ nanocoating. As a result of glutathione coincubation, the viability of tumor cells cultured on the VO_2_ nanocoating was clearly improved, as evidenced by the live/dead staining results shown in **Figure** **6**.

## 3 Discussion

Tumor cells must maintain intracellular redox homeostasis for an exuberant metabolism. Elementary vanadium is highly redox‐active, since it has a series of chemical valence states.^28^ The cellular uptake of the vanadium species by tumor cells may impair their intracellular redox homeostasis and interrupt their normal physiological actions. Therefore, as a redox‐active intermediate oxide, VO_2_ may be able to upset the redox homeostasis of tumor cells.^29^ Meanwhile, the cytotoxicity mechanism of different metal‐containing nanoparticles (metal, metal oxide, semiconductors) is closely related to the release of corresponding toxic ions.^30^

One crucial parameter correlating strongly with metal oxide nanoparticle toxicity is the dissolution of metal ions.^31^ The ionic index (*Z* ^2^/*r*) can directly indicate the dissolution rate of metal ions of nanoparticles, where *Z* and *r* are the charge number and ionic radius (pm), respectively, of the metal cation in the nanoparticles.^32^ For insoluble VO_2_ nanoparticles, the *Z* ^2^/*r* can be estimated at ≈0.13 pm^−2^, which reflects the availability of vanadium ions for dissolution in aqueous solution,^33^ which can be used for local slow vanadium release and delivery.

**Scheme 1:**
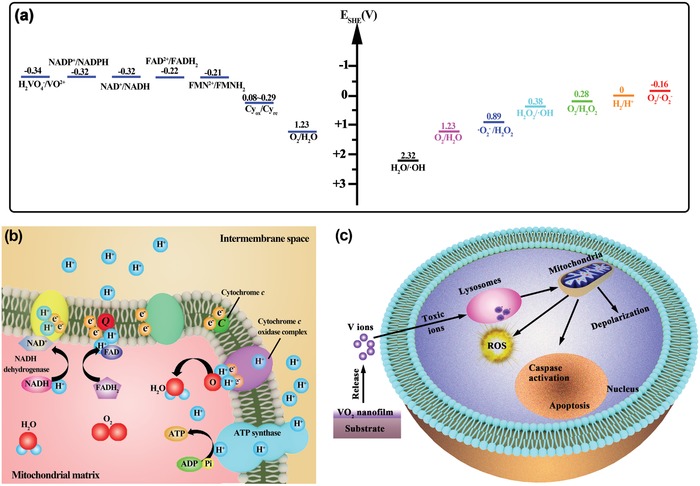
Schematic illustration of anticancer mechanism of VO_2_ nanocoating against tumor cells: a) Redox potentials for the redox couples in mitochondrial electron transfer chain and ROS‐associated redox couples, indicating the feasibility of mitochondrial electron transport chain interruption and the availability of intracellular ROS production. b) Diagram of the mitochondrial electron transport chain and associated interactions, in which electron leakage is able to elevate intracellular ROS production, thereby leading to mitochondria dysfunction. c) Mitochondria‐targeting anticancer process of the VO_2_ nanocoating platform by releasing vanadate ions to cause mitochondria depolarization and oxidative damage, thereby resulting in tumor cell apoptosis.

Here, we first attempt to understand the mechanism of the electron transfer chain on the inner mitochondrial membrane from the perspective of redox chemistry. In the mitochondria, the electron transport proceeds down the redox potentials, as listed in **Table** **1**. Briefly, the electrons are transferred by a series of redox couples (electron carrier proteins) down the electron transport chain. Cytochromes transfer electrons to the cytochrome oxidase complex. The cytochrome oxidase complex then transfers electrons to oxygen, the terminal electron acceptor, and water is formed as the product (**Scheme** **1**a,b). According to the conversion equation for the standard hydrogen electrode (*E*_SHE_, V) and absolute potential level (*E*, eV),^34^ *E*_SHE_ = −4.44 −*E*, the mitochondria‐associated redox potentials are in the range of the biological redox potential (−4.12 to −4.84 eV).^32^ H_2_VO_4_^−^ anions are the most common intracellular form in biological systems, and they can be actively transported into mammalian cells.^35^ The most common forms of vanadium oxidation states are +3, +4, and +5 valences.^36^ The redox potential for the H_2_VO_4_^−^/VO^2+^ redox couple is −0.34 V at pH = 7.^37^ According to the redox potentials of redox couples in the electron transport chain (Table 1), the +5 valent vanadate (H_2_VO_4_^−^) tends to be reduced to the +4 valent VO^2+^ (vanadyl) by the nicotinamide adenine dinucleotide phosphate/reduced nicotinamide adenine dinucleotide phosphate (NADP^+^/NADPH), nicotinamide adenine dinucleotide/reduced nicotinamide adenine dinucleotide (NAD^+^/NADH), flavin adenine dinucleotide/reduced flavin adenine dinucleotide (FAD^2+^/FADH_2_), and flavin mononucleotide/reduced flavin mononucleotide (FMN^2+^/FMNH_2_) redox couples,^38^ according to the following equation: H_2_VO_4_^−^ + 4H^+^ + e^−^ → VO^2+^ + 3H_2_O.^39^

**Table 1:** The redox potentials for redox couples in the electron transfer system of cell mitochondria^69^

Half‐reaction	Redox couple	Redox potentiala) (*E*_0_) [V]
O_2_ + 4H^+^ + 4e^−^ → 2H_2_O	O_2_/H_2_O	1.23
Cytochromes_ox_ + e^−^ → Cytochromes_re_	Cytochromes_ox_/Cytochromes_re_	0.29–0.08
FMN + 2H^+^ + 2e^−^ → FMNH_2_	FMN^2+^/FMNH_2_	−0.21
FAD + 2H^+^ + 2e^−^ → FADH_2_	FAD^2+^/FADH_2_	−0.22
NAD + 2H^+^ + 2e^−^ → NADH + H^+^	NAD^+^/NADH	−0.32
NADP + 2H^+^ + 2e^−^ → NADPH + H^+^	NADP^+^/NADPH	−0.32

^a)^ pH = 7.0.

Based on the above analysis, by destructive extraction of electrons from the mitochondrial membrane, the bioreduction of vanadate tends to interrupt the electron transport chain and impair mitochondrial function. Mitochondria are the major source of ROS in most mammalian cell types, and they convert 1–2% of systemically consumed oxygen to ROS under normal conditions, accounting for the production of 60–80% of H_2_O_2_ in cells.^40^ In this situation, cells can self‐regulate to maintain redox homeostasis through a redox couple defense. However, in pathological conditions, the intracellular ROS level can be elevated through the inhibition of mitochondrial electron transfer chain complexes.^41^ On the other hand, the above‐formed VO^2+^ has the potential to induce the production of hydroxyl radicals (^•^OH) through a Fenton‐like reaction (VO^2+^ + H_2_O_2_ + H^+^ → VO^3+^ + H_2_O +^•^OH).^38^ According to the data listed in **Table** **2**, other types of ROS may also be induced by VO^2+^. For example, by directly reacting with O_2_, VO^2+^ could induce the production of superoxide anion radicals (^•^O_2_^−^), according to the following equation: VO^2+^ + O_2_ + 3H_2_O → H_2_VO_4_^−^ + 4H^+^ +^•^O_2_^−^.^42^ Therefore, the exposure of tumor cells to the VO_2_ nanocoating eventually caused the elevation of intracellular ROS levels, as shown in Figure 4a,c.

**Table 2:** The redox potentials for reactive oxygen species (ROS) associated redox couples^70^

Half‐reaction	Redox couple	Redox potentiala) (*E*_0_) [V]
O_2_ + e^−^ →^•^O_2_^−^	O_2_/•O_2_^−^	−0.16
^•^O_2_^−^ + 2H^+^ + e^−^ → H_2_O_2_	•O_2_^−^/H_2_O_2_	0.89
H_2_O_2_ + H^+^ + e^−^ → H_2_O +^•^OH	H_2_O_2_/•OH	0.38
H_2_O + h^+^ →H^+^ +^•^OH	H_2_O/•OH	2.32
O_2_ + 2H^+^ + 2e^−^ → H_2_O_2_	O_2_/H_2_O_2_	0.28

^a)^ pH = 7.0.

Under normal conditions, cells can maintain redox homeostasis by self‐regulating their ROS level. For this purpose, reduced glutathione (GSH) plays a central detoxification role in cell biology by donating electrons (2GSH → GSSG + 2H^+^ + 2e^−^) in cellular defense against xenobiotics and endogenic toxicants that cause oxidative stress.^43^ On this basis, it is assumed that upon VO_2_‐nanocoating exposure, coincubation with exogenous GSH may be able to alleviate/eliminate the oxidative stress. To verify this, a complementary study using GSH coincubation was conducted. As shown in Figure 5a, after coincubation with GSH, the DCF green fluorescence intensities for intracellular ROS were significantly reduced for all groups, demonstrating that exogenous GSH coincubation could effectively alleviate intracellular ROS levels and help cells maintain redox homeostasis. Quantitatively, after GSH coincubation, no significant differences existed among the intracellular ROS levels of various groups (Figure 5c), providing strong evidence for the elevated production of intracellular ROS upon VO_2_‐nanocoating exposure.

Based on the above, the inhibition of the mitochondrial respiratory chain and the elevation of intracellular ROS production caused mitochondrial impairment. Considering that the mitochondrial depolarization was mainly caused by the elevated ROS production, it was inferred that exogenous GSH coincubation could repair mitochondrial function to some degree. Indeed, as evidenced in Figure 5b, GSH coincubation was able to alleviate the mitochondrial depolarization that occurred in the tumor cells cultured on the VO_2_ nanocoating, indicated by the significant increase in the red fluorescence of the JC‐1 aggregates in the mitochondria. The quantitative results shown in Figure 5d further confirmed these observations. As a result, tumor cell viability was significantly improved by adding GSH, as seen in Figure 6.

Therefore, VO_2_ nanocoating on a surface can regulate the release of vanadate ions to produce the mitochondria‐targeting anticancer activity. To date, the exact molecular mechanism of cell apoptosis is not completely understood, but these studies reveal that a number of key events involved in apoptosis occur in mitochondria.^44^ As an early hallmark of cell apoptosis, the collapse of the mitochondrial membrane potentials (ΔΨ_m_) will cause the depolarization of mitochondria, opening of permeability transition pores, and release of cytochrome c from the mitochondria.^45^ The release of cytochrome c plays a crucial role in activating proapoptotic enzymes, such as caspases.[[qv: 40a,46]] Finally, the tumor cells gradually lose viability due to exposure to the VO_2_ nanocoating. This mitochondria‐targeting vanadate release strategy and oxidative stress‐mediated tumor cell apoptosis process are illustrated in Scheme [qv: 7]. VO_2_ nanocoating can release vanadate ions that purposely target mitochondria in order to disrupt electron transfer and elevate ROS production based on redox chemistry, which then causes mitochondrial depolarization and dysfunction to activate proapoptotic caspases and induce cellular apoptosis. This holds great promise for cancer cell inhibition.

Protein–surface interaction is the initial process occurring at the contact of implant–biological ambiance, and the human serum proteins tend to adsorb to biomaterials. Metal oxides (e.g., TiO_2_, Ta_2_O_5_, Nb_2_O_5_, ZrO_2_) have commonly been used as biocompatible coatings for medical implants.^47^ The surface physicochemical properties of metal oxide coatings play a key role in protein adsorption. The most relevant surface properties identified include surface hydrophobicity/hydrophilicity and the surface charge. On a hydrophobic surface, the driving force for protein adsorption is generally the hydrophobic interaction between the surface and the outside hydrophobic shell of most proteins; meanwhile, on a hydrophilic surface, the driving force for protein adsorption is normally the electrostatic interaction between the surface and the proteins. On the one hand, VO_2_ coatings exhibit a hydrophilic character, with a water contact angle between 20° and 70°.^48^ The adsorption of serum albumin on Ti, Ta, Nb, and Zr oxide films deposited by magnetron sputtering is highly influenced by the surface hydrophilicity or hydrophobicity. Normally, serum albumin adsorption increases with surface hydrophobicity, and the highest albumin adsorption is observed on the hydrophobic surface.^49^ On the other hand, VO_2_ coatings are negatively charged at the physiological pH (7.4), as verified by the surface zeta potentials in Figure 2a. It has been demonstrated that protein adsorption depends on both the surface charges of metal oxide coatings and the protein charges due to the electrostatic interactions between them.^50^ The adsorption of negatively charged serum albumin is more pronounced on positively charged metal oxide surfaces (e.g., Al_2_O_3_) than on negatively charged metal oxide surfaces (e.g., TiO_2_); metal oxide surfaces with negative zeta potential show little or even no protein adsorption.^51^ Further, fibronectin adsorption is decreased on the surface of positively charged metal oxides (e.g., Al_2_O_3_) but higher on the surface of negatively charged metal oxides (e.g., V_2_O_5_, Nb_2_O_5_, TiO_2_). This leads to the observation that osteoblasts stay away from Al_2_O_3_ regions, as better cell adhesion morphology and cytoskeletal arrangement are found in the other regions.^52^ Meanwhile, fibronectin displays higher affinity for more hydrophilic surfaces, which thus can enhance fibronectin adsorption.^53^ In addition, cell functions (cell adhesion, spreading, and cytoskeleton organization) can be greatly enhanced on a fibronectin‐adsorbed hydrophilic surface compared with a fibronectin‐adsorbed hydrophobic surface.^54^ These considerations may account for the lack of significant differences between VO_2_ groups and VO‐0 in initial cell adhesion within 24 h (Figure 3a). It has already been demonstrated that protein adsorption can affect the rate of metal ion release^55^ and may accelerate the release of metal ions.[[qv: 55b,56]] Therefore, further studies are needed to clarify the correlation between protein adsorption and V ion release from VO_2_ nanocoating. With the adsorption of serum proteins, ZnO and NiO nanoparticles can release Zn^2+^ and Ni^2+^ ions, exhibiting strong cytotoxicity on human lung carcinoma A549 cells,[qv: 57] which may support the observed antitumor effect of VO_2_ nanocoating. Finally, it should be pointed out that while our work has manifested the potential of VO_2_ nanocoatings on implant surfaces to resist the growth of tumor cells, this kind of application is more long‐term, and additional testing is required to identify the properties of VO_2_ nanocoating and its influence on cellular behavior in complex environments (e.g., nonlimited cell number, presence of human serum) and over longer periods of time.

Regarding the potential toxicity of vanadium, this issue was studied and found to be dosage dependent. Vanadate has a mitogenic effect on osteoblasts and their precursor cells, and a low concentration of vanadium can even promote DNA and protein (collagen and noncollagen tissue) synthesis in bone, thereby stimulating bone cell proliferation and osteogenesis in vitro.^58^ Furthermore, in vivo studies suggested that controlled vanadium release can accelerate fracture healing,^59^ improve implant–soft tissue integration in wound healing,^60^ and promote bone regeneration in the presence of bacterial infection.[[qv: 58c]] Hence, vanadium can be regarded as “nontoxic” if the dosage is controlled at a low level.[qv: 18] Indeed, a human can tolerate a total of ≈1 mg of vanadium on average.[[qv: 18a]] As shown in Figure [qv: 2]c, vanadium ions were slowly released from the surfaces of VO_2_ nanocoating samples in a time‐dependent and dose‐dependent manner. The number of released ions of each sample is far below the tolerance of the human body. The proliferation and viability results in Figure S1 (Supporting Information) also show that the VO_2_ nanocoating had no significant cytotoxicity on rat bone mesenchymal stem cells, especially for VO‐1 and VO‐2. However, the organic vanadium compound VO(maltol)_2_, which generates vanadate under physiological conditions, has shown the ability to discriminate between human hepatoma cells and normal hepatocytes.^61^ Importantly, the dosage is an important factor in maintaining antitumor‐cell growth and normal cell viability (e.g., mesenchymal stem cells). The present study suggests that the VO‐2 group may be a candidate for future clinical applications.

Furthermore, the main source of potential vanadium toxicity is exposure to high loads of vanadium oxides in the breathing air through the respiratory or gastrointestinal systems. For example, this may occur in industrial enterprises involving vanadium processing. Most dietary vanadium can be excreted by the human body. The vanadium compounds found in the bloodstream are mainly vanadate and vanadyl bound to transferrin. Bony tissue can also store vanadate through blood circulation. Figure S2 (Supporting Information) briefly summarizes the whole process of uptake, distribution, and excretion of vanadium compounds in the human body. Since almost all of the vanadium will be excreted in the form of insoluble VO(OH)_2_ prior to resorption, health risks due to vanadium overload are unlikely to occur. There is currently no clinical evidence proving that vanadium compounds are toxic if administered in a sensible dosage.[[qv: 18b]] As noted by Paracelsus, “all substances are poisons; it is solely the dose that differentiates between a poison and a remedy.”[qv: 62] The current study suggests that the deposition of VO_2_ nanocoating on biomaterial/implant surfaces could induce the death of cancer cells. A notable example is the use of arsenic trioxide (As_2_O_3_) in the treatment of acute promyelocytic leukemia.^63^ Since then, this protocol has been extended to other cancer treatments clinically,^64^ although white arsenic is highly toxic.

## 4 Conclusion

To improve the surface functionalization of implantable biomedical devices for human cancer treatments, a thickness‐tunable VO_2_ nanocoating was first deposited onto the substrate through customized reactive magnetron sputtering. The concept of the mitochondria‐targeting death of tumor cells induced by the controlled release of vanadium ions was proven using human cholangiocarcinoma cells. The results suggest that the released vanadium ions can disrupt the mitochondrial electron transport chain and therefore elevate the intracellular ROS level of tumor cells, thereby leading to mitochondria depolarization due to the collapse of mitochondrial membrane potential. The dysfunction of the mitochondria ultimately induces the apoptosis of the cancer cells. These findings may contribute to the exploitation of VO_2_ nanocoating as an antitumor surface material for implantable biomaterials/devices for cancer treatments.

## 5 Experimental Section

*Sample Fabrication*: Considering float glass contains elements like Na, K, Ca, Mg, etc., which may interfere with the experimental results, fused quartz (consisting of Si and O) was chosen as deposition substrate for the VO_2_ thin films in this study. Homogeneous VO_2_ thin films of a high quality were fabricated using a reactive magnetron sputtering apparatus (ULVAC Corp., Model ACS‐4000‐C4) with high‐purity vanadium metal target of 3 inch and O_2_ gas of 15 sccm at a radio frequency power of 200 W. Prior to growth process of VO_2_ thin films, deposition chamber was pumped down to 10^−4 ^Pa and high pure argon gas (99.999%) was introduced at 25 sccm. During deposition process, substrates were maintained at 450 °C to improve film crystallinity and rotated along vertical axis at a speed of 10 rpm to improve film homogeneity. After the deposition of VO_2_ thin films, samples were cooled down spontaneously to ambient temperature. The thicknesses of VO_2_ thin films were determined using an F20 thin‐film analyzer (FILMSTRICS Corp.). Prepared VO_2_ thin films with thicknesses of 30, 80, and 120 nm were denoted as VO‐1, VO‐2, and VO‐3, respectively. Meanwhile, quartz glass was used as a control (VO‐0).

*Sample Characterization*: AFM (Nanocute SII scanning probe microscope) was used to examine the surface morphology of the prepared VO_2_ thin films, operated in tapping mode under ambient conditions. An XRD (Rigaku Ultima IV), fitted with Cu Kα radiation (λ = 1.541 Å) at a voltage of 40 kV and current of 40 mA, was used to investigate the crystalline structure of the VO_2_ thin films. The XRD patterns were recorded in the range of 20°–80° (2θ) with a step size of 0.02° and a scanning rate of 2° min^−1^. For the XRD test, the glancing incidence angle was fixed at 1°. Phase identification was performed with the help of the Joint Committee on Powder Diffraction Standards (JCPDS) database. Surface chemical components and the chemical state were analyzed by XPS (ESCALAB 250, Thermo Scientific, USA).

*Zeta Potential Measurement*: A SurPASS electrokinetic analyzer (Anton Paar GmbH, Graz, Austria) equipped with a special cell for coating/film was used to measure the surface zeta potentials (ζ) of samples VO‐0, VO‐1, VO‐2, and VO‐3 (size 20 mm × 10 mm × 1 mm). The samples were fixed on holders with double‐sided adhesive tape. An electrolyte of 0.9% NaCl aqueous solution served as measuring medium. The HCl and NaOH solutions were added to adjust the pH value. To measure streaming potential, electrolyte medium was pumped to flow along a pair of sample surfaces and the potentials resulting from ion motion in diffusion layer were recorded based on Helmholtz–Smoluchowski equation

(1) $$\zeta \;\;=\;\;\frac{dU}{dP}\;\;\times \;\;\frac{\eta }{\varepsilon \;\;\times \;\;\varepsilon_{0}}\;\;\times \;\;K$$

where ζ is zeta potential; d*U*/d*P* is slope of streaming potential versus pressure; η is electrolyte viscosity; ε_0_ is vacuum permittivity; ε is dielectric constant of electrolyte; *K* is conductivity. These parameters were collected automatically from SurPASS control and evaluation software and gave the ζ values.

*Vanadium Release Measurement*: The release of the vanadium ions from the VO_2_ nanocoating samples immersed in Roswell Park Memorial Institute 1640 (RPMI 1640; Sigma‐Aldrich) culture medium was determined by inductively coupled plasma optical emission spectrometry (Perkin Elmer, Optima 2100DV). The testing samples were positioned in sterile microcentrifuge tubes (15 mL) filled with 10 mL of fresh RPMI 1640 culture medium, followed by 1, 4, 7, and 14 days of sequential incubation at 37 °C in static conditions. At the end of each incubation period, all the leachates were collected for measurement and the microcentrifuge tubes were supplemented with fresh RPMI 1640 culture medium for subsequent incubation.

*Cancer Cell Culture*: The human cholangiocarcinoma cell line was purchased from the American Type Culture Collection. The tumor cells were initially maintained in the culture medium provided by the supplier in a humidified atmosphere with 5% CO_2_ at 37 °C. Afterward, the cells were passaged at a ratio of 1:2–1:3 every 2–4 days according to the cellular state in RPMI 1640 culture medium supplemented with 10% fetal bovine serum (FBS) and 1% penicillin–streptomycin.

*Cell Viability Assay*: The tumor cell growth and viability were measured by AlamarBlue assay (Sigma‐Aldrich).^65^ The cells were seeded onto VO‐0, VO‐1, VO‐2, and VO‐3 samples in a new 24‐well plate at a density of 5 × 10^4^ cells per well. Five samples were tested for each group. At the end of each culture duration of 1, 4, and 7 days, the supernatant culture medium was removed, and 500 µL of fresh culture medium with 10 v/v% of AlamarBlue reagent was added into each well. After a subsequent 4 h of incubation at 37 °C, 100 µL of culture medium was transferred into a new 96‐well plate to measure the absorbance of the reduced AlamarBlue at wavelengths of 570 and 600 nm on a microplate reader (DTX 800 Series Multimode Detectors, Beckman Coulter). All operating procedures followed manufacturer's instructions.

*Cell Morphology Observation*: The tumor cell morphology and cell–material interactions were further investigated using SEM. At day four of culture, each group of samples was carefully transferred into a new 24‐well plate and gently rinsed with PBS thrice, followed by fixing with 2.5% glutaraldehyde solution overnight at 4 °C. Afterward, all of the samples were sequentially dehydrated in a series of ethanol solutions (30, 50, 75, 90, 95, and 100 v/v%) for 10 min each. Finally, dehydration was performed in absolute ethanol twice and then completed using a series of hexamethyldisilazane ethanol solutions. Prior to SEM observation, the samples were sputter coated with platinum.

*Live/Dead Fluorescence Staining*: The tumor cell viability was further investigated using the fluorescence‐based Live/Dead Staining Kit (Sigma‐Aldrich) following the manufacturer's instructions. In brief, the cells were seeded onto the VO‐0, VO‐1, VO‐2, and VO‐3 samples in a 24‐well plate at a density of 5 × 10^4^ cells per well followed by a subsequent four days of culturing at 37 °C. To stain for cellular viability, calcein‐AM (green fluorescent dye) and propidium iodide (PI, red fluorescent dye) were used to stain the live and dead cells, respectively. The calcein‐AM and PI dyes were diluted to the final concentrations of 2 and 5 × 10^−6^ m in PBS, respectively. After carefully transferring the samples into a new 24‐well plate and washing them gently in PBS thrice, 100 µL of the prepared dye solutions were added into each well of a sample followed by 15 min of incubation at 37 °C. Afterward, both the live and dead cells were visualized by confocal laser scanning microscopy (CLSM; Leica SP8, Germany).

*Mitochondrial Membrane Potential Detection*: As an early marker of cell apoptosis, the mitochondrial membrane potentials of the tumor cells cultured on the VO‐0, VO‐1, VO‐2, and VO‐3 samples were detected by the cationic fluorescent probe JC‐1. JC‐1 aggregates in the mitochondria, indicated by red fluorescence, at a high ΔΨ_m_ (negative inside), while at low ΔΨ_m_, the JC‐1 monomer exists in the cytoplasm and fluoresces green.^66^ The ΔΨ_m_ JC‐1 Assay Kit was purchased from Sigma‐Aldrich. All of the operation procedures strictly followed the manufacturer's instructions. To visualize mitochondrial damage, the cells were seeded on the VO‐0, VO‐1, VO‐2, and VO‐3 samples in a 24‐well plate at a density of 5 × 10^4^ cells per well and then were cultured in a 5% CO_2_ incubator at 37 °C for four days. Prior to use, the JC‐1 staining solution (1 µg mL^−1^ of working solution) was freshly prepared by diluting a 200× stock solution of JC‐1 in warm (37 °C) RPMI 1640 culture medium supplemented with 10% FBS. Then the JC‐1 staining solution was gently added into each well followed by 20 min of incubation at 37 °C. The cells were gently washed twice with the JC‐1 stain buffered solution at 4 °C (ice bath) and then observed immediately using CLSM. The JC‐1 fluorochrome was excited at 488 nm, and the light emission was collected at 570–600 nm for red fluorescence (JC‐1 aggregate) and at 515–545 nm for green fluorescence (JC‐1 monomer). All image acquisition and analyses were carried out using the Leica LAS AF software. The laser beam intensity and photodetector sensitivity were kept constant in order to compare the relative fluorescence intensities between the various groups. Further quantitative analysis was performed by trypsinizing the cells stained with JC‐1 followed by reading the fluorescence intensities on a microplate reader (DTX 800 Series Multimode Detector). The results were expressed as a relative intensity (%) of green and red fluorescence.

*Intracellular Reactive Oxygen Species Determination*: The oxidative stress levels in the tumor cells were investigated using the Intracellular ROS Assay Kit (Sigma‐Aldrich) that contains DCFH‐DA as a fluorescent probe. The DCFH‐DA is able to penetrate the cell membrane where it is then deacetylated into the nonfluorescent DCFH by intracellular esterases. The DCFH remains in cells and can be oxidized into the highly fluorescent DCF by intracellular ROS.^67^ All of the procedures followed the manufacturer's instructions. In brief, the cells were seeded onto the VO‐0, VO‐1, VO‐2, and VO‐3 samples in a 24‐well plate at a density of 5 × 10^4^ cells per well followed by four days of culturing at 37 °C in a 5% CO_2_ incubator. Afterward, the cells were gently washed thrice with PBS. Then the DCFH‐DA was diluted to a final concentration of 10 × 10^−6^ m of staining solution using FBS‐free RPMI 1640 culture medium. Subsequently, the above DCFH‐DA staining solution was added into each well followed by 20 min of incubation at 37 °C and then observed immediately using CLSM. The DCF fluorochrome was excited at 488 nm and light emission was collected at 535 nm. All image acquisition and analyses were carried out using the Leica LAS AF software. The laser beam intensity and photodetector sensitivity were kept constant in order to compare the relative fluorescence intensities between various groups. Further quantitative analysis was conducted by trypsinizing the cells stained with DCFH‐DA and DAPI (cell nuclei staining) followed by reading the fluorescence intensities on a microplate reader (DTX 800 Series Multimode Detectors). The intracellular ROS levels were obtained from the fluorescence reading of DCF and were normalized to the number of tumor cells.

*Glutathione Antioxidant Investigation*: To explore the potential role of oxidative stress in the VO_2_‐induced cell apoptosis, the tumor cells were coincubated with 10 × 10^−3^ m of GSH, the major endogenous antioxidant, to attenuate/eliminate the contribution of oxidative stress in the cellular response to VO_2_‐nanocoating exposure. This strategy has previously demonstrated the effective alleviation of the oxidative stress induced by cellular pro‐oxidants.^67^, ^68^ In brief, the GSH reagent (Sigma‐Aldrich) was freshly dissolved in PBS to prepare a 30 × 10^−3^ m stock solution, sterile filtered, and then diluted in RPMI 1640 culture medium to prepare a 10 × 10^−3^ m working solution. Afterward, the cells were immediately seeded onto the VO‐0, VO‐1, VO‐2, and VO‐3 samples in a 24‐well plate using the freshly prepared cell culture medium containing 10 × 10^−3^ m GSH followed by four days of culturing at 37 °C in a 5% CO_2_ incubator. After that, the following assays were performed according to the manufacturer's instructions. The tumor cell viability after four days of coincubation with 10 × 10^−3^ m of GSH antioxidant on various VO_2_ samples was further investigated using the fluorescence‐based Live/Dead Cell Staining Kit. The mitochondrial membrane potentials of the cultured tumor cells after four days of coincubation with 10 × 10^−3^ m of GSH antioxidant on various VO_2_ samples were further investigated using the fluorescence‐based ΔΨ_m_ JC‐1 Assay Kit. The intracellular ROS levels in the cells after four days of coincubation with 10 × 10^−3^ m of GSH antioxidant on various VO_2_ samples were further investigated using the fluorescence‐based DCFH‐DA Assay Kit.

*Statistical Analysis*: Statistically significant differences (*P* values) between various groups were measured using one‐way analysis of variance and Tukey's multiple comparison tests using a GraphPad Prism 5 statistical software package. All of the data were expressed as a mean ± standard deviation. A value of *P* < 0.05 was considered to be statistically significant and denoted as “*”, *P* < 0.01 was “**”, and *P* < 0.001 was “***”.

## Conflict of Interest

The authors declare no conflict of interest.

## Supporting information

Supplementary
