# Supplementary material for: Vanadium Dioxide Nanocoating Induces Tumor Cell Death through Mitochondrial Electron Transport Chain Interruption
Source: Glob Chall. 2018 Dec 3;3(3):1800058. doi: 10.1002/gch2.201800058 (PMC6436600; doi:10.1002/gch2.201800058)
Supplement: Supplementary file 1 — Supplementary [file GCH2-3-1800058-s001.pdf]

Copyright WILEY-VCH Verlag GmbH & Co. KGaA, 69469 Weinheim, Germany, 2018.

Global Challenges
Open Access

Supporting Information

for *Global Challenges,* DOI: 10.1002/gch2.201800058

Vanadium Dioxide Nanocoating Induces Tumor Cell Death through Mitochondrial Electron Transport Chain Interruption

Jinhua Li, Meng Jiang, Huaijuan Zhou, Ping Jin, Kenneth M. C. Cheung, Paul K. Chu, and Kelvin W. K. Yeung**

Copyright WILEY-VCH Verlag GmbH & Co. KGaA, 69469 Weinheim, Germany, 2018.

Supporting Information

Vanadium Dioxide Nanocoating Induces Tumor Cell Death Through Mitochondrial Electron Transport Chain Interruption

Jinhua Li, Meng Jiang, Huaijuan Zhou, Ping Jin, Kenneth M. C. Cheung, Paul K. Chu *, and Kelvin W. K. Yeung *

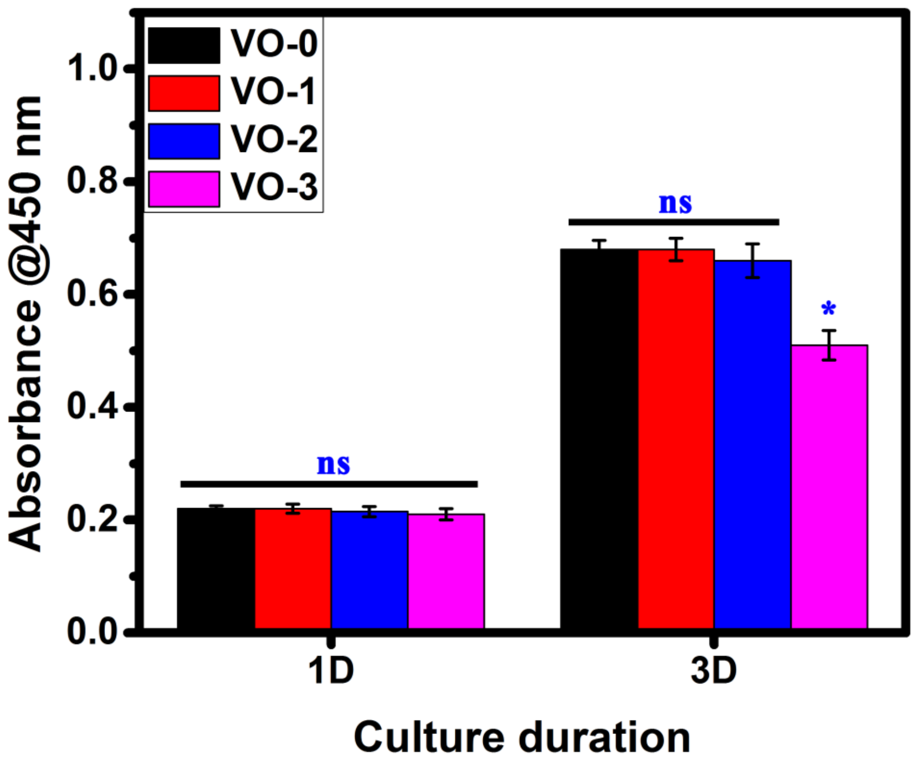

Figure S1. Proliferation and viability results of rBMSCs after cultured on the surfaces of samples VO-0, VO-1, VO-2, and VO-3 for 1 day and 3 days. Note: ns, not significant; $^{*}P < 0.05$ versus VO-0, VO-1 or VO-2.

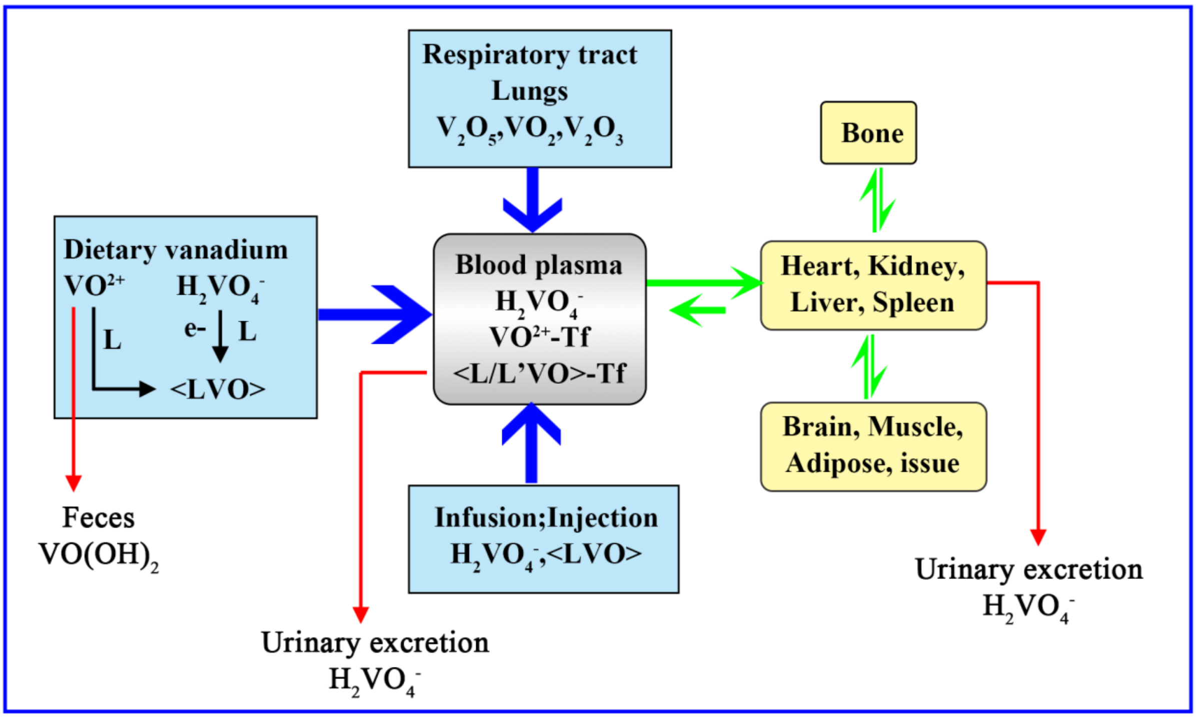

Figure S2. A summary of uptake, distribution and excretion of vanadium compounds in human body. The uptake routes are indicated by broad arrows, whereas the excretion routes are highlighted by broken arrows. The distribution routes are presented by standard arrows and equilibrium arrows, respectively. Tf = transferrin; L is any ligand provided by the nutritional matrix or in a medicinally applied vanadium compound; L′ is a low molecular mass ligand presented in blood serum; and L/L′VO is the abbreviation for a VO^{2+} complex with L and/or L′. Reproduced with permission.[1] Copyright 2013, Springer Science+Business Media Dordrecht.

In Vitro Cytocompatibility Evaluation

The rat bone mesenchymal stem cells (rBMSCs) were used to evaluate the cytocompatibility of the VO_2 nanocoating, and the cell viability was determined using Cell Counting Kit-8 (CCK-8, Beyotime Bio-Tech) assay.[2] Five samples were used for each group, positioned in a new 24-well plate with cell seeding density of 10^5 cells/ml. After 1 day and 3 days of incubation at 37 °C in a humidified environment with 5% CO_2, the culture media were removed and fresh media containing 10% CCK-8 were added into the wells for additional 3 hours of incubation. Finally, 100 μl of the incubated medium from each group was sucked up in a new 96-well plate and the absorbance was measured at 450 nm on a microplate reader.

References

[1] D. Rehder, in Interrelations between Essential Metal Ions and Human Diseases, Vol. 13 (Eds: A. Sigel, H. Sigel, R. K. O. Sigel), Springer Netherlands, 2013, 139.

[2] J. Li, J. Wen, B. Li, W. Li, W. Qiao, J. Shen, W. Jin, X. Jiang, K. W. K. Yeung, P. K. Chu, Advanced Science **2017**, 5, 1700678.
